# Inhibition of mTORC1 induces loss of E-cadherin through AKT/GSK-3β signaling-mediated upregulation of E-cadherin repressor complexes in non-small cell lung cancer cells

**DOI:** 10.1186/1465-9921-15-26

**Published:** 2014-02-26

**Authors:** Eun Young Kim, Arum Kim, Se Kyu Kim, Hyung Jung Kim, Joon Chang, Chul Min Ahn, Yoon Soo Chang

**Affiliations:** 1 Department of Internal Medicine, Yonsei University College of Medicine, 50-1, Yonsei-ro, Seodaemun-gu, Seoul 120-752, Republic of Korea; 2 Biomedical Research Center, Yonsei University College of Medicine, 211 Eonju-ro, Gangnam-gu, Seoul 135-720, Republic of Korea

**Keywords:** mTOR, mTORC1, mTORC2, E-Cadherin repressor complexes, Raptor, Rictor, EMT

## Abstract

**Background:**

mTOR, which can form mTOR Complex 1 (mTORC1) or mTOR Complex 2 (mTORC2) depending on its binding partners, is frequently deregulated in the pulmonary neoplastic conditions and interstitial lung diseases of the patients treated with rapalogs. In this study, we investigated the relationship between mTOR signaling and epithelial mesenchymal transition (EMT) by dissecting mTOR pathways.

**Methods:**

Components of mTOR signaling pathway were silenced by *sh*RNA in a panel of non-small cell lung cancer cell lines and protein expression of epithelial and mesenchymal markers were evaluated by immunoblotting and immunocytochemistry. mRNA level of the E-cadherin repressor complexes were evaluated by qRT-PCR.

**Results:**

IGF-1 treatment decreased expression of the E-cadherin and rapamycin increased its expression, suggesting hyperactivation of mTOR signaling relates to the loss of E-cadherin. Genetic ablation of rapamycin-insensitive companion of mTOR (Rictor), a component of mTORC2, did not influence E-cadherin expression, whereas genetic ablation of regulatory-associated protein of mTOR (Raptor), a component of mTORC1, led to a decrease in E-cadherin expression at the mRNA level. Increased phosphorylation of AKT at Ser473 and GSK-3β at Ser9 were observed in the Raptor-silenced NSCLC cells. Of the E-cadherin repressor complexes tested, Snail, Zeb2, and Twist1 mRNAs were elevated in raptor-silenced A549 cells, and Zeb2 and Twist1 mRNAs were elevated in Raptor-silenced H2009 cells. These findings were recapitulated by treatment with the GSK-3β inhibitor, LiCl. Raptor knockdown A549 cells showed increased expression of N-cadherin and vimentin with mesenchymal phenotypic changes.

**Conclusions:**

In conclusion, selective inhibition of mTORC1 leads to hyperactivation of the AKT/GSK-3β pathway, inducing E-cadherin repressor complexes and EMT. These findings imply the existence of a feedback inhibition loop of mTORC1 onto mTORC2 that plays a role in the homeostasis of E-cadherin expression and EMT, requiring caution in the clinical use of rapalog and selective mTORC1 inhibitors.

## Background

mTOR, a serine/threonine protein kinase regulating cell growth and proliferation, transcription, and protein synthesis, is frequently hyperactivated in neoplastic conditions, tuberous sclerosis complexes (TSC), and lymphangioleiomyomatosis (LAM) [1]. mTOR combines with other proteins to form two different complexes, mTOR complex 1 (mTORC1) and mTORC2. mTORC1 comprises mTOR, mLST8/GβL, regulatory-associated protein of mTOR (Raptor), and PRAS40, while mTORC2 is composed of mTOR, mLST8/GβL, rapamycin-insensitive companion of mTOR (Rictor), mSin1, and Protor [2,3]. mTORC1 is activated by various growth factors and nutrients, and phosphorylates ribosomal S6 kinase1 (S6K1) and eIF-4E-binding proteins (4E-BP1 and 4E-BP2), leading to translation initiation and protein synthesis. mTORC2 regulates Akt and serum/glucocorticoid regulated kinase 1 (SGK1), controlling cell survival and proliferation [2,4].

**Figure 1:**
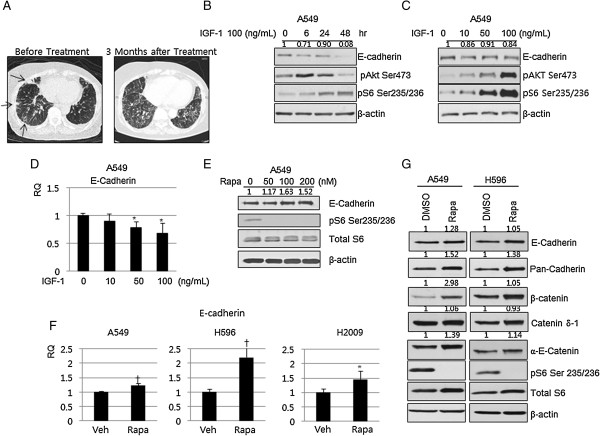
**Involvement of the mTOR pathway in the expression of E-cadherin and components of the adherens junctional complex. (A)** Representative chest computed tomographic imaging of 67-years old female patient, who had taken rapalog for 3 months under the diagnosis of metastatic recurred breast cancer. Compared with imaging prior to the rapalog treatment (left), size of multiple metastatic nodules (arrows) were decreased but ground glass opacity with interstitial thickenings were newly developed throughout the lung fields (right). **(B)** A549 cells were treated with 100 ng/mL IGF-1 for the indicated time, and expression of E-cadherin was evaluated by immunoblotting. A549 cells were treated with the indicated dose of IGF-1 for 16 hr and expression of E-cadherin was evaluated by immunoblotting **(C)** and qRT-PCR **(D)**. **(E)** A549 cells were treated with the indicated dose of rapamycin for 16 hr and expression of E-cadherin was evaluated by immunoblotting. **(F)** A549, H596, and H2009 NSCLC cells were treated with 100 nM rapamycin for 16 hr and E-cadherin mRNA was evaluated by qRT-PCR. **(G)** A549 and H596 NSCLC cells were treated with 100 nM of rapamycin for 16 hr and expression of the components of the adherens junction complex were evaluated by immunoblotting. Data was analyzed by one-way ANOVA followed by Tukey’s multiple comparison test **(D)** or an independent sample *t*-test **(F)**. β-actin was used as a loading control. RQ: relative quantitation, Rapa: rapamycin, veh: vehicle. Error Bars, SD of 3 independent experiments; *, P < 0.05; †, P < 0.01.

There are clinical evidences suggesting deregulation of the mTOR pathway may be involved in the epithelial-mesenchymal transition (EMT). LAM, a rare disease characterized by functional loss of the TSC2 gene leading to aberrant hyperactivation of the mTOR pathway, exhibits loss of E-cadherin expression and uncontrolled expression of smooth muscle actin, implying that the mTOR pathway may be involved in EMT [5]. Interstitial lung disease and nephritis, which are frequently observed during treatment with mTOR inhibitors in solid organ transplant recipients and cancer patients, may be another clues indicating relationship between mTOR pathway and EMT (Figure 1A) [6,7].

Two mechanisms of rapamycin-induced pneumonitis have been proposed: direct alveolar cytotoxicity and immune-mediated toxicity [6]. The direct alveolar cytotoxicity hypothesis is based on the observation that the incidence of interstitial pneumonitis is dose-dependent, whereas the immune-mediated toxicity hypothesis is based on the wide range of time courses observed and the wide range of trough serum levels of rapamycin. However, mechanistic explanations of these hypotheses are elusive. Detailed identification of the underlying mechanisms of mTOR inhibitor-induced EMT requires dissecting the inherent function of each mTOR complex with analysis of the pharmacodynamics and pharmacokinetics of mTOR inhibitors.

One possible explanation relating EMT and mTOR pathway would be disruption of feedback regulation of Akt by mTORC1. IGF-1 mediates upregulation of the E-cadherin transcriptional repressors, Snail and Slug, and pharmacologic inhibition of mTOR inhibits IGF-1-induced E-cadherin loss [8]. Akt is activated either by phosphorylation at Thr 308 by phosphoinositide-dependent kinase 1 (PDPK1) or at Ser473 by mTORC2 [4]. The feedback inhibition of Akt by mTORC1 is mediated by destabilization of IRS1 [9,10] or Grb10 [11]. mTOR inhibitors have been tested in clinical trials against cancer and for management of LAM, but the feedback activation of the PI3K-Akt pathway, which occurs with mTORC1 inhibition, might had lessen their clinical utility [11-14].

In this study, we sought to determine the effect of each mTORC on the expression of E-cadherin. To achieve this, we selectively deleted Raptor and Rictor, the regulatory complexes of mTORC1 and mTORC2, respectively. Decreased E-cadherin was observed in Raptor-silenced cells whereas no change in E-cadherin was observed in Rictor and TSC2-silenced cells. Increased phosphorylation of Akt and GSK-3β was observed in Raptor-deleted cells leading to up-regulation of E-cadherin repressor complexes. These findings suggest that selective inhibition of mTORC1 would lead to EMT, which might be the underlying mechanism of intestinal pneumonitis and nephritis in patients treated with rapalogs.

## Materials and methods

### Antibodies, cells, and plasmids

A549 cells were purchased from ATCC (Manassas, VA, USA), and 293FT cells were purchased from Invitrogen (Gaithersburg, MD, USA). H2009, H596, and H1650 cells were obtained from the Korean Cell Line Bank (Seoul, Korea). pLKO.1-Raptor 1 *sh*RNA (plasmid #1857), −Raptor 2 *sh*RNA (plasmid #1858), −Rictor 1 *sh*RNA (plasmid #1853), −Rictor 2 *sh*RNA (plasmid #1854), −TSC2 *sh*RNA (plasmid #15478), psPAX2 (plasmid #12260), and pMD2. G (plasmid #12259) were obtained from Addgene (Cambridge, MA, USA). Antibodies, unless otherwise stated, were obtained from Cell Signaling Technology and are listed in Additional file 1: Table S1 (Danvers, MA, USA).

### Patients’ characteristics

Clinical information of the 305 patients, who had taken rapalogs under the diagnosis of neoplastic diseases (n = 198) or solid organ transplantation recipient (n = 107) between September 2009 and September 2013, was retrospectively reviewed. Patients’ diagnoses are as follows: renal cell carcinoma (n = 151), thyroid cancer (n = 21), malignant lymphoma (n = 10), neuroendocrine carcinoma (n = 7), hepatocellular carcinoma (n = 5), breast cancer (n = 2), and rectal cancer (n = 2). All the solid organ transplantation recipients had taken rapamycin under the diagnosis of kidney recipient (Table 1). Rapalog-induced interstitial pneumonitis was identified by radiologic diagnosis of chest CT images with exclusion of pulmonary infection or other cause of pulmonary infiltration. Chest CT scan was performed at the onset of respiratory symptoms and signs, not routinely serial follow up exam. Inclusion criteria for drug-induced interstitial pneumonitis of chest CT images were followed by the suggestion of Endo *et al*. [15]. The use of clinical information received exemption from IRB deliberation.

**Table 1:** Clinical characteristics of the rapalogs induced interstitial pneumonitis

	**Medication**	**No. of cases**	**No. of interstitial pneumonitis (%)**	**Grade 3-4 (%)**	**Time to development (months)**
Neoplastic disease	Everolimus	107	7 (6.5)	3 (2.8)	5.8
	Temsirolimus	91	4 (4.4)	1 (1.1)	4.6
Solid organ transplantation	Rapamycin	107	5 (4.7)	3 (2.8)	8.6

### *sh*RNA and *si*RNA experiments

Lentiviral *sh*RNAs were generated as described elsewhere [16]. Cells transduced with pLKO.1 lentiviral vector were maintained in RPMI medium containing 5% fetal bovine serum. Puromycin (1.0 μg/mL) was added for A549 and H1650 cells, while concentrations of 2.0 μg/mL and 3.0 μg/mL were used for H460 and H1299 cells, respectively.

### Western blotting

Cells were harvested using 2X LSB lysis buffer containing protease inhibitor cocktail and phosphatase inhibitor cocktail (Sigma, St. Louis, MO, USA) on ice. After sonication, BCA protein assay reagent (Thermo Scientific, Rockford, IL, USA) was used for protein quantification. Protein lysates (30–50 μg) were separated by gel electrophoresis on 7.5% to 12% polyacrylamide gels and analyzed by western blot using nitrocellulose membranes (Bio-Rad Laboratories, Inc., Richmond, CA, USA). Expression level of each protein was measured using ImageJ (http://imagej.nih.gov/ij/) and quantified relative to that of β-actin.

### Immunocytochemistry

5 × 10^5^ cells were plated in 6-well plates containing a sterilized coverslip. The next day, cells were fixed with 4% formaldehyde in PBS, incubated in blocking solution containing 5% BSA in PBS, and then anti-E-cadherin mouse (1:100) and anti-Vimentin rabbit antibodies (1:200) were added for 16 hr. The next day, cells were washed and anti-mouse IgG (Alexa Fluor 555 conjugate) and anti-rabbit IgG (Alexa Flour 488 conjugate, Cell Signaling Technology) secondary antibodies were added. Nuclei were counterstained with DAPI (1:1000, Sigma) in PBS and imaged using an LMS510 confocal microscope (Carl Zeiss, Oberkochen, Germany).

### Quantitative real time (qRT)-PCR

Total RNA was extracted using TRI Reagent® (Ambion, Austin, TX, USA). Reverse transcription and quantitative PCR were performed using High Capacity cDNA reverse transcriptase and TaqMan® FastUniversal PCR Master Mix (2X), respectively. qRT-PCR analysis was conducted using TaqMan® gene expression assay reagents, and the StepOnePlus Real-Time PCR system (Applied Biosystems, Carlsbad, CA, USA) with an inventoried primer-probe set described in Additional file 2: Table S2. IPO8 was amplified in a primer-limited fashion using primers labelled with VIC dye as an endogenous control.

### Statistical analysis

Data were analyzed either by one-way ANOVA followed by Tukey’s multiple comparison test or an independent sample *t*-test. All statistical analyses were two-tailed.

## Results

### Involvement of the mTOR pathway in the loss of E-cadherin expression

During the recent 4 years, 16 (5.2%) out of 305 patients, who had taken rapalogs under the diagnosis of either neoplastic diseases or solid organ recipient, experienced clinically apparent interstitial lung disease in the study institutes (Figure 1A and Table 1). Among them, 7 patients showed severe interstitial pneumonitis (grade ≥ 3). The mean time to development of interstitial pneumonitis was 6.4 months (5.8 months for everolimus, 4.6 for temsirolimus, and 8.6 for rapamycin). Together with the loss of E-cadherin expression in the LAM, development of interstitial pneumonitis during rapalogs treatment brought us a question whether aberration mTOR signaling impacts the expression of E-cadherin and EMT.

To explore this, A549 cells were treated IGF-1 and expression of E-cadherin was evaluated by immunoblotting. Treatment with IGF-1 decreased E-cadherin expression in time- and dose-dependent experiments, and E-cadherin expression was more dependent on time, reaching a nadir at 48 hr after IGF-1 treatment (Figure 1B and C). There was a dose-dependent decrease in E-cadherin mRNA with IGF-1 treatment, suggesting that the loss of E-cadherin occurred at the transcriptional level (Figure 1D). Next, NSCLC cells were treated with rapamycin, an mTOR inhibitor, and expression of E-cadherin was evaluated by immunoblotting. Treatment with rapamycin clearly increased E-cadherin protein and mRNA expression (Figure 1E,F and G). We also examined the effect rapamycin on the expression of other components of the adherens junction complex by immunoblotting. Inhibition of the mTOR pathway with rapamycin increased expression of E-cadherin, β-catenin, catenin-δ-1, and α-E-catenin (Figure 1G). Taken together, these findings suggest that the mTOR pathway is involved in regulating the expression of E-cadherin and the other components of adherens junction complex.

### Disruption of mTORC1 activity by transduction with Raptor *sh*RNA inhibits E-cadherin expression

**Figure 2:**
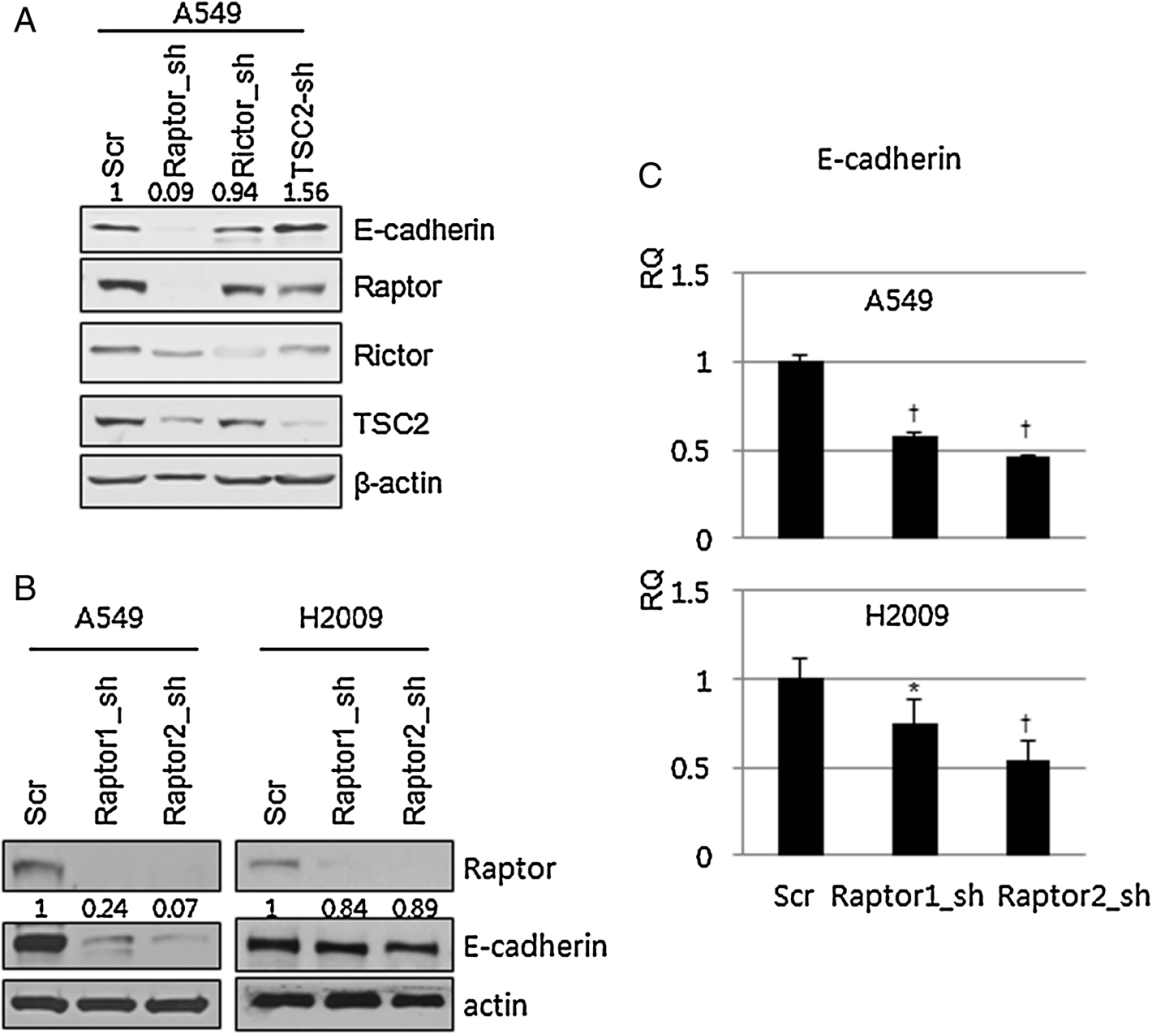
**Disruption of mTORC1 suppresses E-cadherin expression. (A)** A549 cells were stably transduced with lentiviral shRNA vectors targeting Raptor, Rictor, or TSC2. Expression of E-cadherin was analyzed by Western blotting. **(B)** A549 and H2009 cells were transduced with 2 different pLKO.1-Raptor-shRNA constructs and expression of E-cadherin was analyzed using Western blotting. **(C)** E-cadherin mRNA was analyzed by qRT-PCR in Raptor-silenced A549 and H2009 cells. Data was analyzed by one-way ANOVA followed by Turkey’s multiple comparison test. β-actin was used as a loading control. Error Bars, SD of 3 independent experiments; RQ, relative quantitation; *, P < 0.05; †, P < 0.01.

Since recent reports indicate that mTOR functions as a part of mTORC1 or mTORC2 depending on its binding partners, and each complex has different inherent roles, we questioned whether loss of E-cadherin is mediated through mTORC1 or mTORC2. To investigate this, A549 cells were transduced with *sh*RNA against Raptor, Rictor, or TSC2, which regulate mTORC1, mTORC2 and Rheb, respectively. Samples were then blotted for E-cadherin. Raptor *sh*RNA-transduced cells lost E-cadherin expression, while Rictor and TSC2 *sh*RNA-transduced cells did not (Figure 2A). To further confirm this finding, different Raptor *sh*RNA constructs were transduced into A549 and H2009 NSCLC cells and the expression of E-cadherin was evaluated by immunoblotting and real-time PCR. E-cadherin expression decreased in A549 and H2009 cells when they were transduced with either Raptor *sh*RNA construct, and the loss was more prominent in A549 cells (Figure 2B). E-cadherin mRNA expression also decreased in A549 and H2009 cells transduced Raptor *sh*RNA (Figure 2C). These findings suggest that inhibiting mTORC1 by silencing Raptor reduces E-cadherin expression at the transcriptional level.

### Aberrant Akt-GSK-3 signaling in Raptor deficient cells

**Figure 3:**
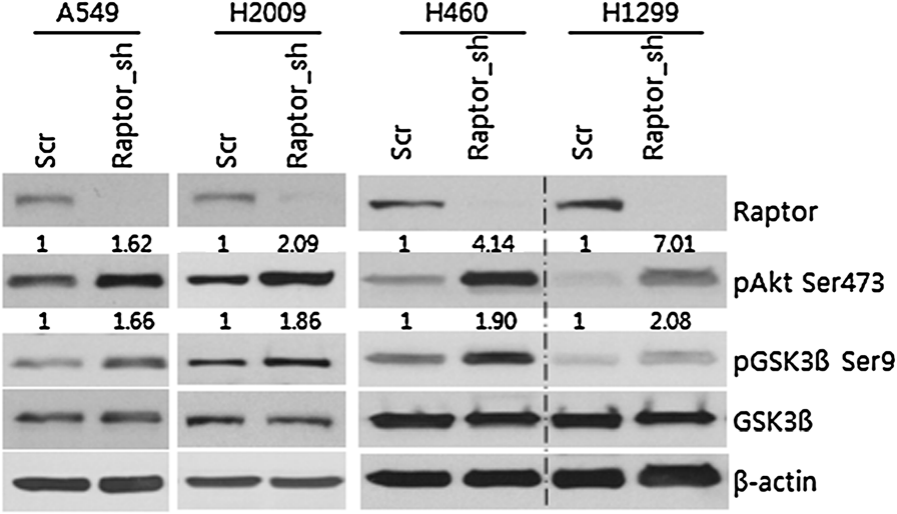
**Aberrant Akt-GSK3β signaling in Raptor-silenced cells.** A549, H2009, H460, and H1299 NSCLC cells were transduced with scrambled or Raptor shRNA and pAKT-Ser473 and pGSK-Ser9 levels were evaluated by Western blotting. β-actin was used as a loading control.

Because feedback regulation of pAkt is one of the functions of mTORC1, we next evaluated the phosphorylation of Akt at Ser473 in Raptor-silenced NSCLC cells. Increased phosphorylation of Akt at Ser473 was observed in A549, H2009, H460, and H1299 Raptor-silenced NSCLC cells, causing inhibitory phosphorylation of the Akt substrate GSK-3β at Ser9 (Figure 3). To further confirm hyperactivation of Akt/GSK-3β signaling in Raptor-silenced NSCLC cells, *si*RNA against Raptor was introduced into A549, H460, H1299, and H2009 NSCLC cells (data not shown). All NSCLC cells treated with Raptor *si*RNA showed increased levels of pAKT-Ser473 and pGSK-Ser9 compared to those transduced with scrambled *si*RNA. These findings suggest that Akt/GSK-3β pathway aberrations are a general finding in cells with mTORC1 disrupted by Raptor silencing and that Akt phosphorylation leads to functional activation of Akt/GSK-3β signaling.

### Transcriptional repressors of E-cadherin are elevated in Raptor-silenced cells

**Figure 4:**
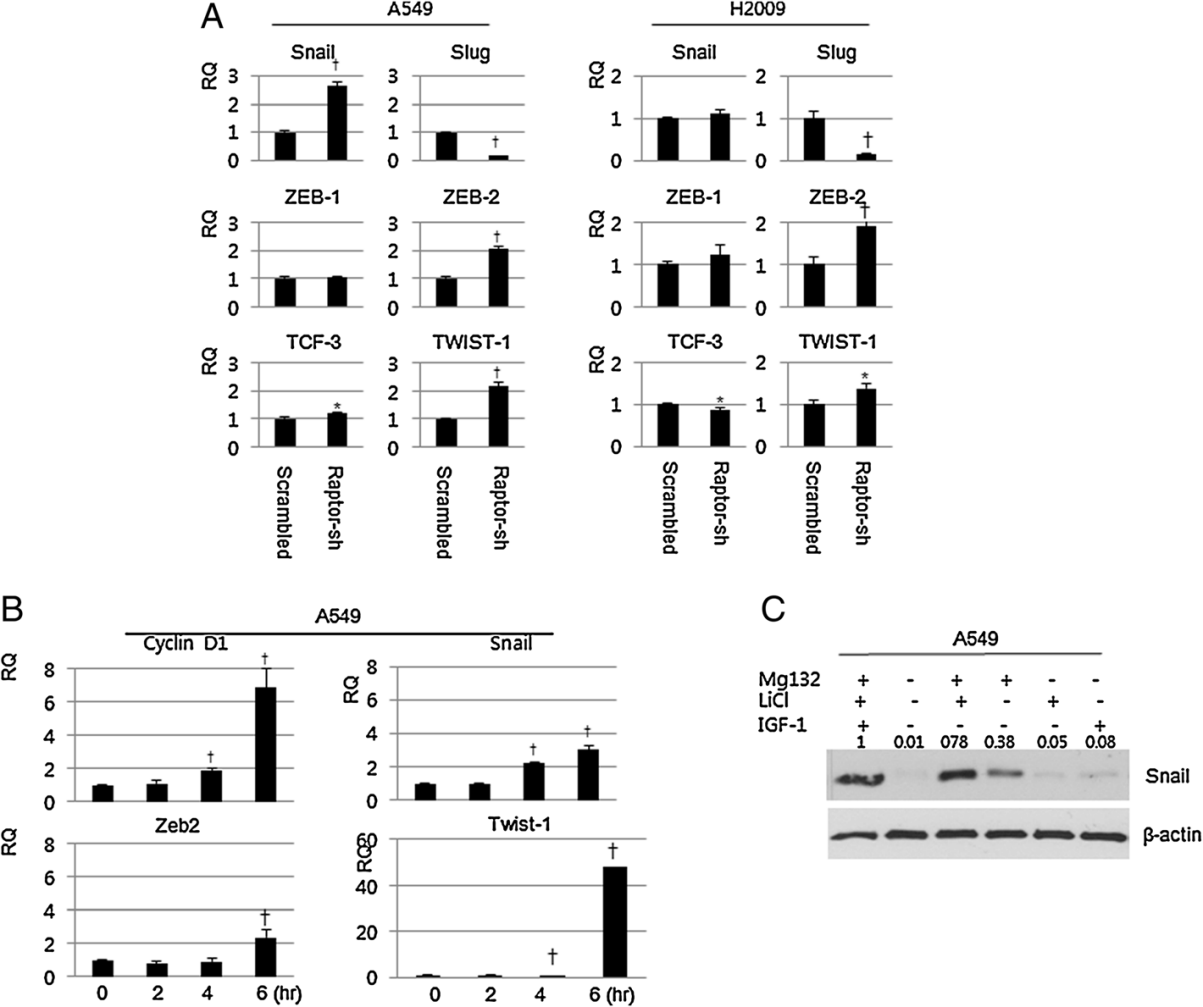
**E-cadherin repressor complexes are elevated in Raptor-silenced cells. (A)** A549 and H2009 cells were transduced with scrambled or Raptor shRNA and E-cadherin repressor complex mRNAs were evaluated by qRT-PCR. Data were analyzed by the *t*-test. **(B)** A549 cells were treated with 40 mM LiCl for the indicated times and E-cadherin repressor complex mRNAs were evaluated by qRT-PCR. Data were analyzed by one-way ANOVA followed by Tukey’s multiple comparison test. **(C)** A549 cells were treated with MG132 (10 μM) and LiCl (40 mM), and/or IGF (10 ng/mL) for 6 hr and expression of Snail was evaluated by immunoblotting. β-actin was used as a loading control. Error Bars, SD of 3 independent experiments; RQ, relative quantitation; *, P < 0.05; †, P < 0.01.

Because transcriptional inhibition of E-cadherin is frequently mediated by E-cadherin repressor complexes and we observed decreased E-cadherin mRNA expression in Raptor-silenced NSCLC cells, we evaluated E-cadherin repressor complex mRNA expression by qRT-PCR. Among the E-cadherin repressor complexes tested, Snail, Zeb2, and Twist1 had increased expression in Raptor-silenced A549 cells and Zeb2 and Twist1 were elevated in Raptor-silenced H2009 cells (Figure 4A). We postulated that inhibitory phosphorylation of GSK-3β at Ser9 by Akt might cause the increase in Snail, Zeb2, and Twist mRNA. To test this, A549 parental cells were treated with LiCl, a GSK-3β inhibitor, and E-cadherin repressor complex mRNA expression was evaluated by qRT-PCR. Indeed Snail, Zeb2, and Twist1 mRNAs were elevated in cells treated with LiCl (Figure 4B). Because Snail is mainly regulated by β-TrCP mediated proteosomal degradation secondary to GSK-3β-mediated phosphorylation [17], we were curious about the contribution of the mRNA level elevation to the expression of Snail protein. LiCl treatment slightly increased the expression of Snail and its effect was comparable to that of IGF-1. Combined treatment with LiCl and MG132 strongly increased expression of Snail. Taken together, these findings suggest involvement AKT/GSK-3β signaling in the loss of E-cadherin repressor complexes in Raptor-silenced cells (Figure 4C).

### EMT in Raptor-silenced NSCLC cells

**Figure 5:**
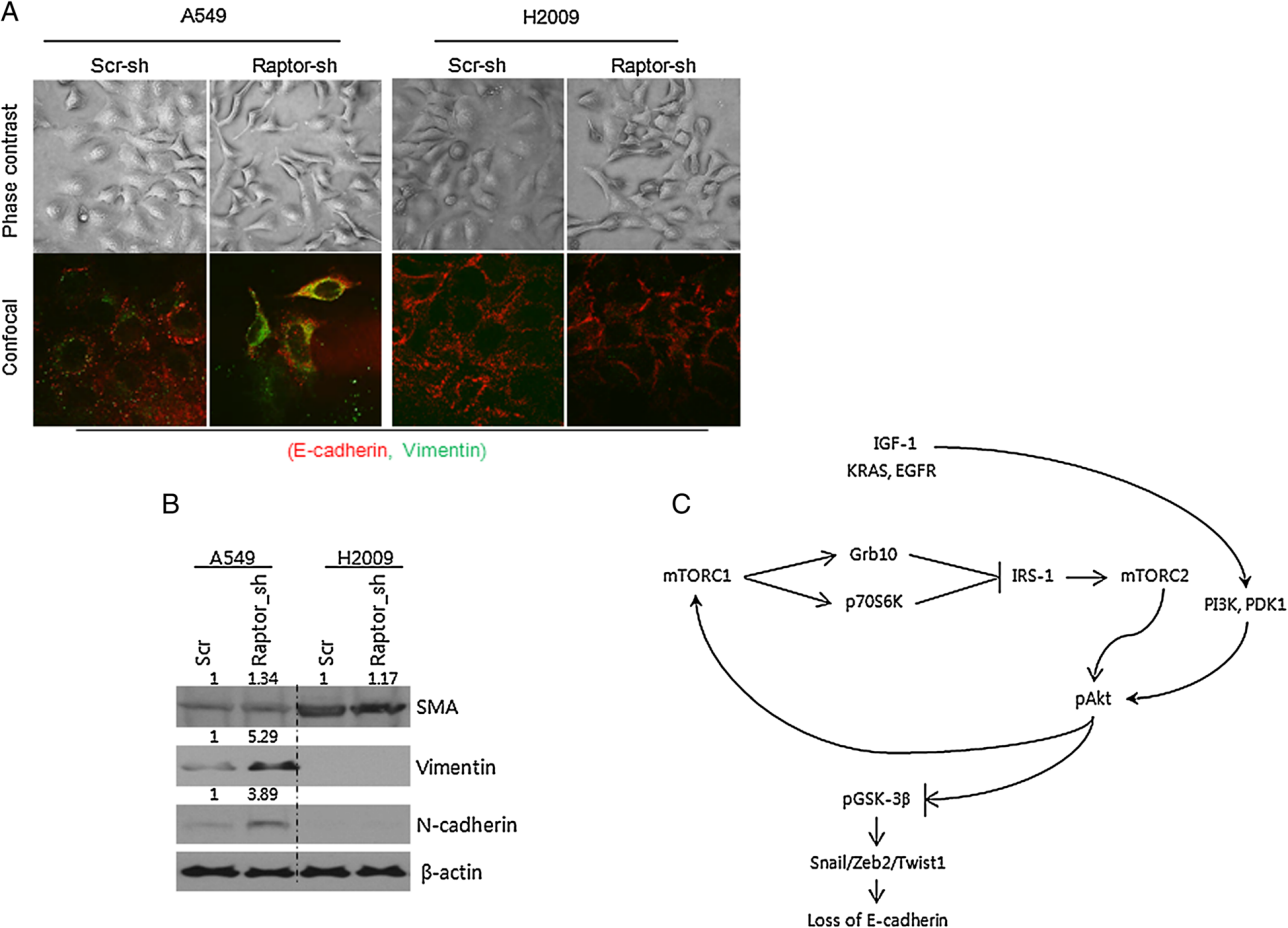
**EMT in Raptor-silenced cells. (A)** Phase contrast and immunocytochemical image of scrambled and Raptor shRNA-transduced A5549 and H2009 cells (X1,000). E-cadherin: Red, Vimentin: Green. **(B)** Expression of EMT markers α-smooth muscle actin (SMA), Vimentin, and N-cadherin were evaluated in scrambled and Raptor shRNA-transduced A549 and H2009 cells by Western blotting. β-actin was used as a loading control. **(C)** Illustration of the role of mTORC1 in the regulation of mTORC2-mediated EMT.

EMT entails morphological and phenotypic changes of epithelial cells leading to loss of cellular polarity and intercellular adhesion, and the development of migratory and invasive properties. Phase-contrast imaging of Raptor-silenced cells shows a transition from the typical cobblestone appearance to elongated spindle shapes. These findings are more prominent in A549 cells than in H2009 cells. To further elucidate the EMT in Raptor-silenced cells, immunocytochemical staining against E-Cadherin and Vimentin was performed. Raptor-silenced A549 cells showed a marked increase in Vimentin and decrease in E-cadherin expression whereas H2009 cells did not exhibit changes in the expression of E-cadherin and Vimentin (Figure 5A). Then mesenchymal markers, α-smooth muscle actin (SMA), Vimentin, and N-cadherin, were blotted in scrambled- and Raptor-*sh*RNA-transduced cells. Similar to the findings in the morphologic study, Raptor-silenced H2009 cells did not show changes in the expression of Vimentin, N-cadherin, and SMA. Expression of Vimentin and N-cadherin was increased in Raptor-silenced A549 cells while that of SMA was unchanged, suggesting that disruption of mTORC1 leads to EMT in a subset of cells (Figure 5B).

## Discussion

Since the discovery of rapamycin in 1975 as an antibiotic, rapamycin and its derivatives, rapalogs, have been widely used as immunomodulators in solid organ transplant patients, for the treatment of LAM [13] and Tuberous Sclerosis Complex [18], and for the treatment of neoplastic conditions such as Kaposi’s sarcoma [19], hepatocellular carcinoma [20], and renal cell carcinoma. Rapamycin also prolonged the life expectancy of mice [21] and explosively broadens its clinical applications. In addition to the common side effects, such as impaired glucose metabolism and changes in lipid profiles, rapalogs can cause interstitial pneumonitis and nephritis.

Pulmonary side effects, including interstitial pneumonitis, bronchiolitis obliterans organizing pneumonia, granulomatous interstitial pneumonitis and rarely, diffuse alveolar damage with alveolar hemorrhage [22-24], are frequent adverse effects of rapalogs and its incidence is 5-15% [25,26]. There are 2 hypotheses for rapamycin-induced pneumonitis: direct alveolar cytotoxicity and immune-mediated toxicity [6]. However, histopathological analysis of rapamycin-induced lung injury, showing accumulation of extracellular matrix collagen and fibroblast proliferation, suggests that EMT could be another mechanism of rapalog-induced lung injury [27].

The downregulation of E-cadherin is a hallmark of EMT and is involved in transcriptional repressors, including the zinc finger proteins, Snail, Slug, Zeb1, and Zeb2, and the basic helix-loop-helix factors, TCF3 and Twist1. Snail and Slug are up-regulated at the transcriptional level by activation of PI3K/Akt/mTOR signaling [8]. They are further controlled by GSK-3β phosphorylation-mediated degradation [17,28]. In our study, silencing of Raptor caused increased phosphorylation of Akt at Ser473 and subsequent inhibitory phosphorylation of GSK3β at Ser9. Raptor-silenced cells showed upregulation of Snail, Zeb2, and Twist1. These findings could be recapitulated by treatment with GSK-3β inhibitor, resulting in profound effects on the mRNA expression of E-cadherin repressor complexes. Among the cells expressing E-cadherin that were evaluated in this study (A549, H596, H1650, and H2009), A549 cells showed the most prominent morphologic and phenotypic changes after Raptor silencing. We postulated these from the characteristics of A549 cells that have a loss of LKB1 which leads dependency to mTORC1.

Comparing to the silencing of Raptor that caused loss of mTORC1 mediated feedback inhibition of pAKT-Ser473 and decrease of E-cadherin expression, rapamycin treatment increased that of E-cadherin. In our time and dose dependent experiments, treatment of rapamycin showed a prompt decrease in pS6 phosphorylation within 10 min and a delayed decrease in phosphorylation of Akt at Ser 473 (Additional file 3: Figure S1). These findings are compatible to the findings from Montero et al. [29] and implies that exposure to the higher dose and/or prolonged exposure to rapamycin inhibits both mTORC1 and mTORC2 leading inhibition of both mTORCs, which resulting inhibition of pAkt-Ser473 and increase of E-cadherin expression. The half-life of rapamycin in whole blood is 135 hr (vs*.* 7.2 hr in plasma) in humans [30]. The usual dose of rapamycin ranges from 1 to 3 mg/day and is adjusted to maintain trough levels of 4 to 12 ng/mL [26]. It is unclear whether repetitive oral administration of 1 to 3 mg/day of rapamycin to adults is mTORC1 selective or influences both mTORCs. These suggest further in vivo studies are required with cautious dosing schedules and optimal dosing ranges.

Our study has a number of limitations. Besides that animal data was not presented, findings in this study were obtained from lung cancer cell lines. When compared to the silencing of the Rictor, which did not result in noticeable changes in cell proliferation, disruption of mTORC1 by Raptor silencing significantly inhibited cell growth and proliferation. Transduction of Raptor *sh*RNA into BEAS-2B cells inhibited cell proliferation and resulted in failure of cell line establishment. This may originate not only from the unique role of each mTOR complex but also from the existence of alternative pathways which circumvent blocking of the each pathway. In other words, relayed signaling through PI3K-PDK1 pathway may be helpful to circumvent the blocking of mTORC2 whereas there is no alternative pathway which helps bypassing blocking of mTORC1. This bypass of mTORC2 blocking led less phenotypic changes in Rictor silenced cells. We also were unable to observe changes in E-cadherin in TSC2-suppressed cells. Because loss of TSC function is still considered a major mechanism that leads to uncontrolled proliferation of LAM cells, further study on this subject is warranted.

## Conclusions

Selective inhibition of mTORC1 induced phosphorylation of AKT Ser473 and GSK-3β Ser9, increasing expression of E-cadherin repressor complexes and decreasing expression of E-cadherin. This finding suggests caution in using selective mTORC1 inhibitors and/or p70S6K1 inhibitors in clinical practice because of the danger of AKT-mediated EMT (Figure 5C).

## Abbreviations

EMT: Epithelial-mesenchymal transition; LAM: Lymphangioleiomyomatosis; mTORC1: mTOR complex 1; mTORC2: mTOR Complex 2; SMA: α-smooth muscle actin; Raptor: Regulatory-associated protein of mTOR; Rictor: Rapamycin-insensitive companion of mTOR.

## Competing interests

The authors declare that they have no competing interests.

## Authors’ contributions

EYK participated in the design of the study, reviewed clinical data, carried out the IRB process, immunocytochemistry, qRT-PCR, and the manuscript draft. AK carried out the molecular genetic studies and data analysis. SKK participated in the study design, statistical assistance, and interpretation of the data. HJK, JC, and CMA participated in the study design. YSC conceived the study, and participated in its design and coordination and helped to draft the manuscripts. All authors read and approved the final manuscript.

## Supplementary Material

Additional file 1: Table S1 List of antibodies used in this study. Click here for file

Additional file 2: Table S2 List of Taqman primers and probes used in this study. Click here for file

Additional file 3: Figure S1 Dose and time effect of rapamycin on the mTOR pathway. A549 cells were treated with vehicle, 1, and 10 nM of rapamycin for the indicated times and expression of pAkt-Ser473 and p-GSK3β Ser9, and pS6 Ser235/236 were evaluated by immunoblotting. Click here for file
