# Supplementary material for: Inhibition of mTORC1 induces loss of E-cadherin through AKT/GSK-3β signaling-mediated upregulation of E-cadherin repressor complexes in non-small cell lung cancer cells
Source: Respir Res. 2014 Feb 26;15(1):26. doi: 10.1186/1465-9921-15-26 (PMC3941688; doi:10.1186/1465-9921-15-26)
Supplement: Additional file 1: Table S1 — List of antibodies used in this study. [file 1465-9921-15-26-S1.pdf]

Supplementary Table 1. List of antibody used in this study.

Number	Name	Company	Cat No
1	Phospho-S6 Ribosomal Protein (Ser235/236) (D57.2.2E) XP® Rabbit mAb	Cell Signaling Technology	4858S
2	S6 Ribosomal Protein (5G10) Rabbit mAb	Cell Signaling Technology	2217S
3	Akt (pan) (C67E7) Rabbit mAb	Cell Signaling Technology	4691S
4	Phospho-Akt (Ser473) (D9E) XP® Rabbit mAb	Cell Signaling Technology	4060S
5	Tuberin/TSC2 (D93F12) XP® Rabbit mAb	Cell Signaling Technology	4308S
6	Rictor (53A2) Rabbit mAb	Cell Signaling Technology	2114S
7	Raptor (24C12) Rabbit mAb	Cell Signaling Technology	2280S
8	β-Catenin (6B3) Rabbit mAb	Cell Signaling Technology	9582S
9	Pan-Cadherin (28E12) Rabbit mAb	Cell Signaling Technology	4073S
10	E-Cadherin (24E10) Rabbit mAb	Cell Signaling Technology	3195S
11	β-Actin Antibody	Cell Signaling Technology	4967L
12	Vimentin (D21H3) XP® Rabbit mAb	Cell Signaling Technology	5741S
13	GSK3-Beta(27C10)	Cell Signaling Technology	9315
14	phospho-GSK3beta(Ser9)	Cell Signaling Technology	9336S
15	Catenin delta-1 antibody	Cell Signaling Technology	4989S
16	alpha-E-Catenin (23B2) Rabbit mAb	Cell Signaling Technology	3240S
17	N-Cadherin Rabbit Ab	Cell Signaling Technology	4061P
18	E-cadherin (67A4)	Santa Cruz Biotech	sc-21791
19	ant-SMA antibody	sigma	4795
20	Alexa-Fluor 488 goat anti-mouse IgG (H+L)	Invitrogen	A11001
21	Alexa-Fluor 633 goat anti-mouse IgG (H+L)	Invitrogen	A21052
