# Supplementary material for: Inhibition of mTORC1 induces loss of E-cadherin through AKT/GSK-3β signaling-mediated upregulation of E-cadherin repressor complexes in non-small cell lung cancer cells
Source: Respir Res. 2014 Feb 26;15(1):26. doi: 10.1186/1465-9921-15-26 (PMC3941688; doi:10.1186/1465-9921-15-26)
Supplement: Additional file 2: Table S2 — List of Taqman primers and probes used in this study. [file 1465-9921-15-26-S2.pdf]

Supplementary table 2. List of Taqman primer and probes used in this study.

Lab No	Gene name	Cat No	gene symbol	Label	Entrez Gene ID
1	IPO8	Hs00183533	IPO8	VIC_Primer Limited	10526
2	E-Cadherin	HS01023894	CDH1	FAM	999
4	cyclin D1	Hs00765553_m1	CCND1	FAM	595
8	ZEB1	Hs00232783_m1	ZEB1	FAM	6935
9	ZEB2	Hs00207691_m1	ZEB2	FAM	9839
10	SNAI1	Hs00195591_m1	SNAI1	FAM	6515
11	SNAI2	Hs00950344_m1	SNAI2	FAM	6591
12	E12/E47	Hs00413032_m1	TCF3	FAM	6929
13	Twist1	Hs01675818_s1	TWIST1	FAM	7291
