# Supplementary figures and images for: Inhibition of mTORC1 induces loss of E-cadherin through AKT/GSK-3β signaling-mediated upregulation of E-cadherin repressor complexes in non-small cell lung cancer cells
Source: Respir Res. 2014 Feb 26;15(1):26. doi: 10.1186/1465-9921-15-26 (PMC3941688; doi:10.1186/1465-9921-15-26)

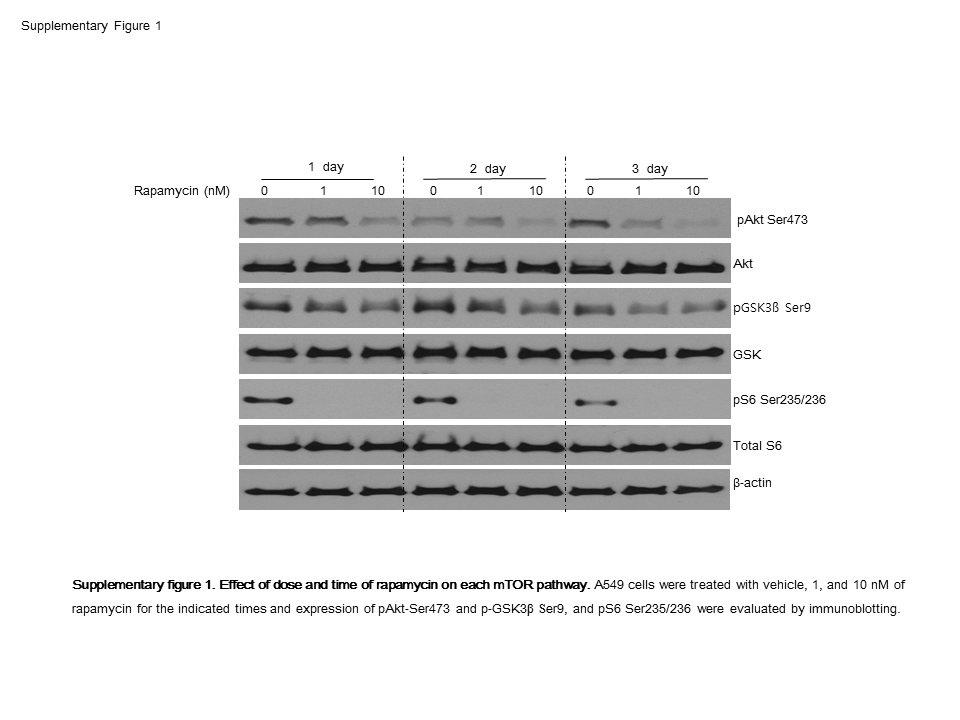

Supplement: Additional file 3: Figure S1 — Dose and time effect of rapamycin on the mTOR pathway. A549 cells were treated with vehicle, 1, and 10 nM of rapamycin for the indicated times and expression of pAkt-Ser473 and p-GSK3β Ser9, and pS6 Ser235/236 were evaluated by immunoblotting. [file 1465-9921-15-26-S3.tiff]
